# Identification of *Helicobacter pylori*‐carcinogenic TNF‐alpha‐inducing protein inhibitors via daidzein derivatives through computational approaches

**DOI:** 10.1111/jcmm.18358

**Published:** 2024-05-02

**Authors:** Jehad Zuhair Tayyeb, Shibam Mondal, Md Anisur Rahman, Swapon Kumar, Imren Bayıl, Shopnil Akash, Md. Sarowar Hossain, Taha Alqahtani, Magdi E. A. Zaki, Jonas Ivan Nobre Oliveira

**Affiliations:** ^1^ Department of Clinical Biochemistry, College of Medicine University of Jeddah Jeddah Saudi Arabia; ^2^ Pharmacy Discipline, School of Life Sciences Khulna University Khulna Bangladesh; ^3^ Department of Pharmacy Islamic University Kushtia Bangladesh; ^4^ Department of Pharmacy Jahangirnagar University Savar Bangladesh; ^5^ Department of Bioinformatics and Computational Biology Gaziantep University Gaziantep Turkey; ^6^ Department of Pharmacy Daffodil International University Dhaka Bangladesh; ^7^ Department of Pharmacology, College of Pharmacy King Khalid University Abha Saudi Arabia; ^8^ Department of Chemistry, College of Science Imam Mohammad Ibn Saud Islamic University Riyadh Saudi Arabia; ^9^ Department of Biophysics and Pharmacology, Bioscience Center Federal University of Rio Grande do Norte Natal Brazil

**Keywords:** ADMET, computer‐aided drug design, DFT, gastric cancer, *Helicobacter pylori*, molecular docking, molecular dynamic simulation

## Abstract

Gastric cancer is considered a class 1 carcinogen that is closely linked to infection with *Helicobacter pylori* (*H. pylori*), which affects over 1 million people each year. However, the major challenge to fight against *H. pylori* and its associated gastric cancer due to drug resistance. This research gap had led our research team to investigate a potential drug candidate targeting the *Helicobacter pylori*‐carcinogenic TNF‐alpha‐inducing protein. In this study, a total of 45 daidzein derivatives were investigated and the best 10 molecules were comprehensively investigated using *in silico* approaches for drug development, namely pass prediction, quantum calculations, molecular docking, molecular dynamics simulations, Lipinski rule evaluation, and prediction of pharmacokinetics. The molecular docking study was performed to evaluate the binding affinity between the target protein and the ligands. In addition, the stability of ligand–protein complexes was investigated by molecular dynamics simulations. Various parameters were analysed, including root‐mean‐square deviation (RMSD), root‐mean‐square fluctuation (RMSF), radius of gyration (Rg), hydrogen bond analysis, principal component analysis (PCA) and dynamic cross‐correlation matrix (DCCM). The results has confirmed that the ligand–protein complex CID: 129661094 (07) and 129664277 (08) formed stable interactions with the target protein. It was also found that CID: 129661094 (07) has greater hydrogen bond occupancy and stability, while the ligand–protein complex CID 129664277 (08) has greater conformational flexibility. Principal component analysis revealed that the ligand–protein complex CID: 129661094 (07) is more compact and stable. Hydrogen bond analysis revealed favourable interactions with the reported amino acid residues. Overall, this study suggests that daidzein derivatives in particular show promise as potential inhibitors of *H. pylori*.

## 1 INTRODUCTION

Gastric cancer (GC), which is classified as a class 1 carcinogen, is associated with *Helicobacter pylori* (*H. pylori*), which affects over 1 million patients annually.^1^ *H. pylori* is a Gram‐negative bacterium that can lead to various diseases, including gastritis, gastrointestinal ulcers, dysplasia and gastric cancer.^2^, ^3^ GC has a higher mortality rate in East and Central Asia and Latin America, regions where it is more common.^4^ Remarkably, *H. pylori* is responsible more than 60% of GC cases and contributes to more than 8.2% of all cancer‐related deaths.^5^ The predicted number of GC diagnoses in 2020 was over 1.1 million, with 770,000 deaths. Incidence rates were higher in men than in women.^6^

The rising antibiotics resistance of *H. pylori* makes current thereapies for GC difficult to treat and a major health concern to the people.^7^, ^8^ The development of new drugs to combat GC and *H. pylori* is therefore urgently needed.

Natural, and synthetic products are now being studied extensively due to their low toxicity and potential anticancer properties. These products have a broad spectrum of pharmacological activity and are found in fruits, vegetables, spices and medicinal plants. Research has documented that natural, and synthetic products have various anticancer properties, including induction of apoptosis in gastric cancer cells, inhibition of metastasis, and inhibition of cell growth.^9^, ^10^

Daidzein is an isoflavone primarily obtained from soy plants. It has a wealth of nutritional and therapeutic benefits, including anticancer properties and the ability to reduce oxidative damage, regulate the immune response and induce cell death. The structural similarities of the isoflavone daidzein are very similar to the human hormone oestrogen^11^, ^12^ Additionally, daidzein is reported to have minimal toxicity, making it an attractive candidate for developing and discovering potential therapeutic agents against *H. pylori*.^13^

We have targeted the *Helicobacter pylori*‐carcinogenic TNF‐alpha‐inducing protein produced by the bacterium *Helicobacter pylori*. This particular protein is responsible for the production of the tumour necrosis factor‐*α* (TNF‐*α*)‐inducing protein (Tip*α* protein), which binds to the cell surface and is involved in the development of GC.^14^, ^15^, ^16^

Conventional drug discovery research is a lengthy and risky process that takes more than 10–15 years and requires a huge amount of resources and funding. The average cost of developing a drug from the laboratory to clinical application is over 1–2 billion dollars.^17^ This study was designed to determine the potential drug candidate against *H. pylori* using daidzein derivatives to accelerate the development of innovative gastric cancer therapies through computational approaches and to speed up drug development while reducing costs. By identifying the most promising biomolecules at an early stage, this approach increases the possibility of successful therapeutic development for GC and avoids costly wet‐laboratory failures.

## 2 COMPUTATIONAL METHOD AND WORKING PROCEDURE

### 2.1 Target structure selection and preparation

**FIGURE 1:**
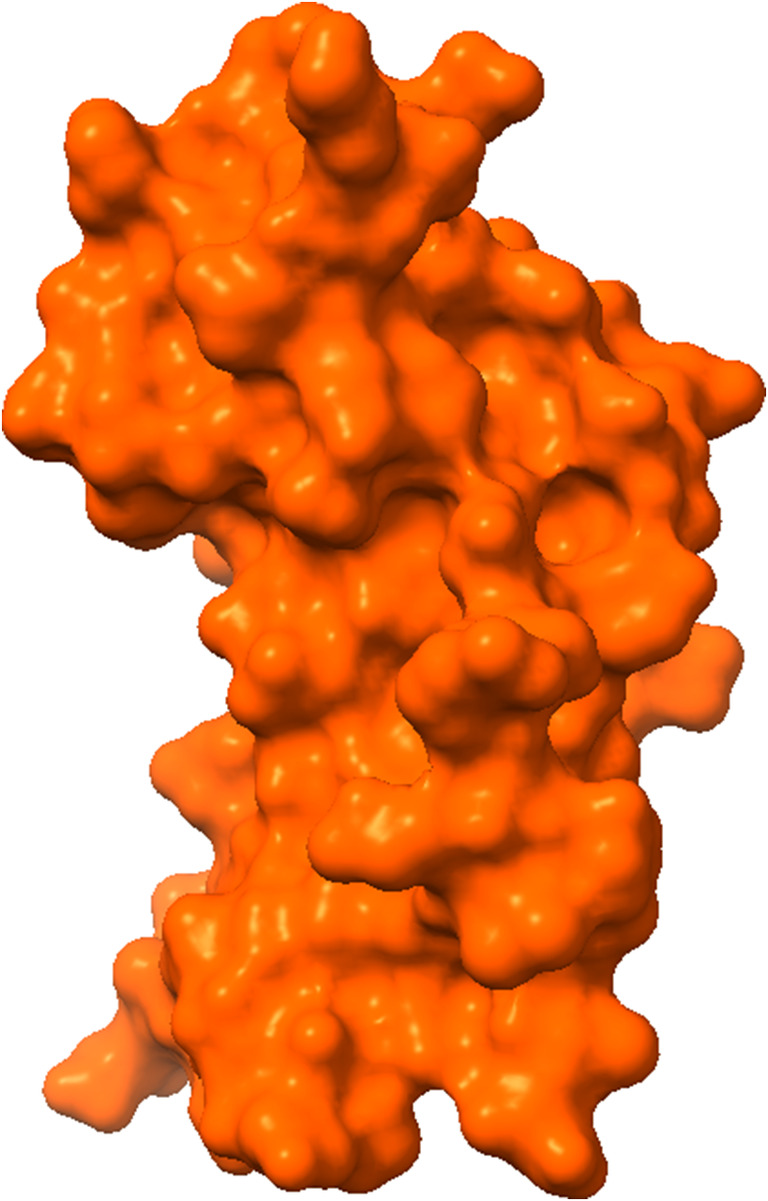
Three‐dimensional protein structure of the targeted protein.

We obtained the 3D structures of *Helicobacter pylori*‐carcinogenic TNF‐alpha‐inducing protein (PDB ID: 3GUQ) (Figure 1) from the Protein Data Bank at https://www.rcsb.org/.^16^ To prepare the protein structures for further analysis, we removed all heteroatoms and water molecules using the program Pymol (version 1.3).^18^ Subsequently, this prepared protein structure was stored in pdb format for molecular docking experiment against target ligands/molecules.

### 2.2 Ligand preparation and optimization

**FIGURE 2:**
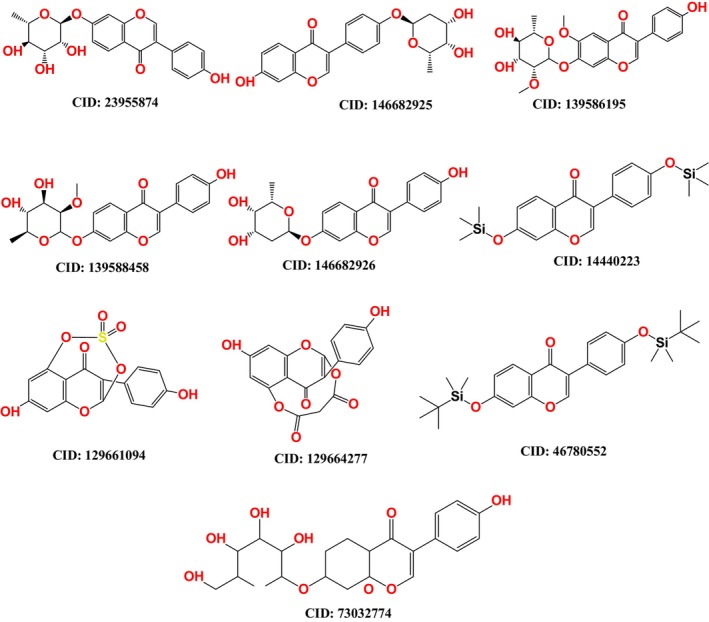
Molecular structure of daidzein derivatives.

One of the most important aspects of molecular docking studies is the preparation of the ligands. This involves rearranging the atoms in the molecule until the configuration with the lowest ground‐state energy is found.^19^, ^20^ This step is essential because molecular docking and molecular dynamics (MD) simulations require ligands with well‐defined geometries. First, we collected the chemical structures of daidzein derivatives from the PubChem database in SDF format (Figure 2).^21^ The structure of each ligand was optimized at the B3LYP/6‐31G(d,p) level using density functional theory (DFT) in Gaussian 09.^22^ After optimization, we calculated the electronic properties such as energy gap, hardness, softness and the energies of the frontier orbitals—the highest occupied molecular orbital (HOMO) and the lowest unoccupied molecular orbital (LUMO). These properties provide about the chemical reactivity properties of the compounds.^23^ Finally, the optimized ligands were saved pdb format to be used for further computational studies such as molecular docking and dynamic simulations. Figure 2 shows the molecular structures.

### 2.3 Molecular docking

Numerous bioinformatic methods have been developed to study and evaluate the molecular interactions between proteins and ligands within their binding sites. In our study, molecular docking was utilized to the development of novel therapeutic compounds targeting the *Helicobacter pylori*‐carcinogenic TNF‐alpha‐inducing protein.^24^

We used the free PyRx package version 0.8 with AutoDock Vina to perform the docking methodology.^25^

To ensure the most accurate docking position with a minimal margin of error, we set the exhaustion parameter of the program. The ligands were considered flexible during docking, while the proteins were treated as rigid. The interactions between chemicals and proteins were further investigated and visualized using the Pymol and Discovery applications.

### 2.4 ADMET, drug‐likeness and Lipinski rule prediction

Predicting the ADMET properties of selected lead compounds during the development of new drugs is invaluable to avoid adverse effects.^26^ This approach can significantly reduce costs by excluding ineffective compounds from the development pipeline. To achieve this, we used the pkCSM web server (https://biosig.lab.uq.edu.au/pkcsm/prediction), which is a graph‐based algorithm to calculate ADMET values for selected compounds and provide possible ADMET properties for these molecules.^27^ This system is known for its precision in assessing the pharmacokinetic and toxicological properties of pharmaceutical substances based on their chemical and structural characteristics. Using the Simplified Molecular Input Line Entry System (SMILES) into the pkCSM search bar and captured the expected ADMET properties.^27^

In addition, we investigated the Lipinski's Rule of Five, a widely recognized analysis for evaluating the pharmacological and biological activity of drug‐like substances.^28^ Therefore, we assessed Lipinski's rule using the SwissADME server (http://www.swiss‐ame.ch/) and investigated the possible drug‐like features.^29^

### 2.5 Molecular dynamic (MD) simulations

The molecular dynamic simulations for the two ligand–protein complexes and the standard amoxicillin with the best docking interactions were performed against the carcinogenic TNF‐alpha‐inducing target protein of *H. pylori* (PDB ID: 3GUQ) using the Gromacs software (version 2020) at 100 ns.^30^ The CHARMM36m force field parameters were used for the simulation. The topology files for both the protein and the ligands were created using the CHARMM‐GUI server to prepare them for the simulation.^31^ During the simulation, the protein–ligand complexes were placed in a rectangular water box with a side length of 10 Å to facilitate solvation. Solvation was achieved by adding TIP3 water molecules under periodic boundary conditions (PBC). Na+ and Cl− ions were added to the systems at a concentration of 0.15 M to maintain the neutrality.

The process of energy minimization was performed out using the steepest‐descent algorithm with 5000 iterations, keeping a tolerance limit of 1000 kJ mol^‐1^ nm^‐1^. Subsequently, the three complex structures were equilibrated at a temperature of 310 K for a duration of 125,000 picoseconds while maintaining the NVT (constant number of particles, volume and temperature) boundary constraints.

The molecular dynamics simulation was performed under NPT (constant number of particles, pressure and temperature) boundary conditions for a duration of 100 nanoseconds at a temperature of 310 Kelvin, with a fixed pressure of 1 atmosphere and a time step of integration set to 2 femtoseconds.

Electrostatic interactions were calculated using the particle‐mesh Ewald (PME) algorithm with a Coulomb cut off value of 1.2 nm.^32^ All bond distances involving hydrogen atoms were constrained during the simulations using the Lincs algorithm. After re‐centring the simulation output for analysis, the trajectory data were analysed sequentially using the VMD program.

### 2.6 Binding free energy calculation using MM‐PBSA

The evaluation of binding free energy for ligands in proteins is an essential parameter of molecular dynamics simulations, and the calculation of free energy play an important role in this determination. In this study, the MM‐PBSA approach was used to determine the binding free energy of interaction between ligands and the *Helicobacter pylori*‐carcinogenic TNF‐alpha‐inducing target protein (PDB ID: 3GUQ). The estimation of the binding free energy (ΔG) was performed using Equation (1) with the script MMPBSA.py from the AMBER package.^33^

(1) ΔGbind=−Gcomplex+Greceptor−Gligand.

The G‐complex stands for the free energy associated with the formation of the complex. Similarly, the G‐receptor denotes the free energy associated with the receptor, while the G‐ligand represents the free energy associated with the ligand.

## 3 RESULT AND DISCUSSION

### 3.1 Pass prediction analysis

Pass prediction is the first step in determining whether a bioactive chemical will be effective or not. The Pa (probability ‘to be active’) and Pi (probability ‘to be inactive’) are described. The probability of success increases as Pa increases and Pi decreases, but Pa and Pi will never be the same or equal. These novel molecular properties should be predicted early stages so that the efficacy of a particular molecule can clearly be understood.^34^, ^35^, ^36^, ^37^ We first collected the pass prediction scores of 45 derivatives of daidzein and then selected the 10 most potent compounds for further investigation based on their maximal antineoplastic properties (Table S1). The ranges of Pa scores for antibacterial 0.600+ for most molecules, and antineoplastic (0.800+), which are much larger than those for antifungal and antiparasitic properties (Table 1). These data are from pass online website ‘http://way2drug.com/PassOnline/predict.php’.^38^

**TABLE 1:** Biological pass prediction spectrum computation of daidzein derivatives.

PubChem ID	Antibacterial		Antiviral (Influenza)		Antineoplastic		Antiparasitic	
	Pa	Pi	Pa	Pi	Pa	Pi	Pa	Pi
23955874	0.606	0.008	0.551	0.017	0.808	0.011	0.527	0.013
146682925	0.622	0.008	0.359	0.061	0.840	0.008	0.712	0.005
139586195	0.626	0.008	0.445	0.033	0.826	0.009	0.431	0.025
139588458	0.633	0.007	0.507	0.022	0.829	0.009	0.561	0.01
146682926	0.622	0.008	0.359	0.061	0.840	0.008	0.712	0.005
14440223	0.217	0.103	—	—	0.921	0.005	0.248	0.075
129661094	—	—	—	—	0.917	0.005	—	—
129664277	0.211	0.108	—	—	0.938	0.004	0.169	0.112
46780552	0.203	0.114	—	—	0.908	0.005	0.457	0.02
73032774	0.570	0.011	0.687	0.006	0.788	0.013	0.325	0.049

### 3.2 Lipinski rule, pharmacokinetics and drug‐likeness

We uploaded selected ligand structure files to the SwissADME website to obtain the required properties and Lipinski rule evaluations. According to the rules, drug‐like molecules should have a molecular weight of less than 500 Daltons. It is evident that the compounds we have selected fulfil this criteria.

Moreover, none of the compounds has more than five hydrogen bond donors and the number of hydrogen bond acceptors does not exceed 10, so the Lipinski rule is fulfilled. We also evaluated the Log *p* values (partition coefficient in octanol/water) for the candidate lead compounds, which are within acceptable ranges.^39^, ^40^

These properties play as an essential role in drug discovery and help to define the drug‐like properties of the ligands. As summarized in Table 2, the drug‐like profiles of all the ligands show promising results.

**TABLE 2:** Data of Lipinski rule and drug‐likeness.

CID	Molecular weight	Hydrogen bond acceptor	Hydrogen bond donor	Consensus Log Po/w	Lipinski rule	
					Result	Violation
23955874	400.38	8	4	1.21	Yes	0
146682925	384.38	7	3	2.00	Yes	0
139586195	444.43	9	3	1.61	Yes	0
139588458	414.41	8	3	1.71	Yes	0
146682926	384.38	7	3	1.97	Yes	0
14440223	398.60	4	0	4.36	Yes	0
129661094	348.28	8	2	1.75	Yes	0
129664277	354.27	8	2	1.84	Yes	0
46780552	482.76	4	0	6.13	Yes	0
73032774	422.43	9	5	0.11	Yes	0

### 3.3 Computational ADMET data prediction

Prediction of ADMET (Absorption, Distribution, Metabolism, Excretion and Toxicity) characteristics is essential in drug development, as over 50% of medications fail in clinical trials due to poor ADMET properties. We used pkCSM web server to predict the ADMET profiles for our selected compounds (Table 3).

**TABLE 3:** ADMET result data listed.

No	Absorption		Distribution	Metabolism		Excretion		Toxicity	
	Water solubility Log S	Human Intestinal Absorption (%)	VDss (log L/kg)	CYP450 1A2 Inhibitor	CYP2D6 substrate	Total Clearance (ml/min/kg)	Renal OCT2 substrate	Max. tolerated dose (log mg/kg/day)	Skin Sensitization
23955874	−3.326	66.577	0.192	No	No	0.172	No	0.404	No
146682925	−3.279	79.183	−0.16	Yes	No	0.228	No	0.105	No
139586195	−3.444	79.649	−0.032	No	No	0.271	No	0.102	No
139588458	−3.404	72.503	−0.054	No	No	0.308	No	0.163	No
146682926	−3.381	82.264	0.119	Yes	No	0.256	No	0.151	No
14440223	−6.21	94.509	0.373	Yes	No	0.254	No	0.324	No
129661094	−3.294	100	0.405	Yes	No	0.268	No	0.115	No
129664277	−3.766	91.455	−0.263	No	No	0.402	No	−0.109	No
46780552	−7.767	93.895	0.415	Yes	No	0.024	No	0.339	No
73032774	−2.53	49.139	0.211	No	No	1.11	No	−0.005	No

We focused on the most important absorption features: ‘water solubility’, and ‘absorption in the human intestine’. Solubility is described by the Log S scale: insoluble: <−10; poor: <−6, moderate: <−4; soluble: <−2; and very high: <0.^41^, ^42^, ^43^ Most of the compounds exhibited good water solubility, with Log S values under −4. Only the compounds CID: 14440223 and CID: 46780552 showed poor solubility (Log S above −4). In addition, all compounds showed excellent absorption rates, with compound CID: 129661094 being the most promising at 100%.

The distribution of the drug in the body is another essential factor that has been used to evaluate the movement/transfer from the bloodstream to the tissues.^44^, ^45^ Volume of distribution (VDss) values of drug is less than −0.15 indicate poor distribution, while values above 0.45 indicate higher distribution.^46^, ^47^ In our study, some compounds reported moderate VDss values, ranging between high and low values, while the other compounds reported low VDss values.

We investigated the metabolism parameters of the compounds, and in that case cytochrome P450 isoforms, CYP450 1A2 and CYP2D6 substrate are considered. All compounds could inhibit CYP450 1A2, while all compounds were negative for the CYP2D6 substrate enzymes. These enzymes are essential for drug metabolism.^48^, ^49^

Total clearance and Renal OCT2 substrate are also essential parameter for the excretion profile of a drug.^50^, ^51^ The compound CID: 129664277 has reported the highest value for total clearance score, while the compound CID: 73032774 has reported the lowest value. None of the compounds were identified as potential OCT2 substrates.

Finally, the maximum tolerated dose (MTD) was analysed. It is used to predict the maximum amount of medication a patient can take in a given period of time.^52^, ^53^ In this study, the compounds CID: 23955874, CID: 14440223 and CID: 46780552 has reported the maximum tolerated dose of 0.404, 0.324 and 0.339 log mg/kg/day, respectively. This indicates that the CID: 23955874, CID: 14440223 and CID: 46780552 could be taken at 0.404, 0.324 and 0.339 log mg/kg/day as the maximum amount (Table 3).

### 3.4 Molecular docking against targeted protein

Molecular docking is one of the most important bioinformatics methods in computer‐aided drug design and enables the investigation of non‐bonded interactions and energy binding in protein‐ligand complexes. Molecular docking provides valuable insights into the interaction and binding affinity of protein–ligand complexes.^54^, ^55^, ^56^

The most notable binding energy observed for the target receptor *Helicobacter pylori*‐carcinogenic TNF‐alpha‐inducing protein (PDB ID: 3GUQ) is −8.5 kcal/mol and −8.9 kcal/mol in the compounds (CID: 129661094 and 129664277), and the ranges of binding affinities are −6.3 kcal/mol to −8.9 kcal/mol (Table 4). For the standard amoxicillin, −7.0 kcal/mol was observed. This indicates that most of the daidzein derivatives have a stronger binding affinity compared to amoxicillin. To evaluate the protein–ligand interactions of the above compounds, we used Pymol v2.4.1, Chimera X software and BIOVIA Discovery Studio.^57^ Finally, the two best compounds and the standard amoxicillin were subjected to molecular dynamics simulation.

**TABLE 4:** Molecular docking score.

No/PubChem CID		*Helicobacter pylori*‐carcinogenic TNF‐alpha‐inducing protein (PDB ID: 3GUQ)
		Binding affinity (kcal/mol)
1.	23955874	−8.1
2.	146682925	−7.8
3.	139586195	−7.7
4.	139588458	−8.1
5.	146682926	−7.9
6.	14440223	−7.3
7.	129661094	−8.9
8.	129664277	−8.5
9.	46780552	−7.0
10.	73032774	−6.3
Standard amoxicillin		−7.0

### 3.5 Protein–ligand interaction and molecular docking poses

The BIOVIA Discovery Studio Visualizer and the Chimera application were used to analyse the molecular interactions between the compounds and the macromolecules of the receptors in more detail. In this case, the two best ligand–protein complexes were selected based on the highest binding affinity to the receptor proteins.

**FIGURE 3:**
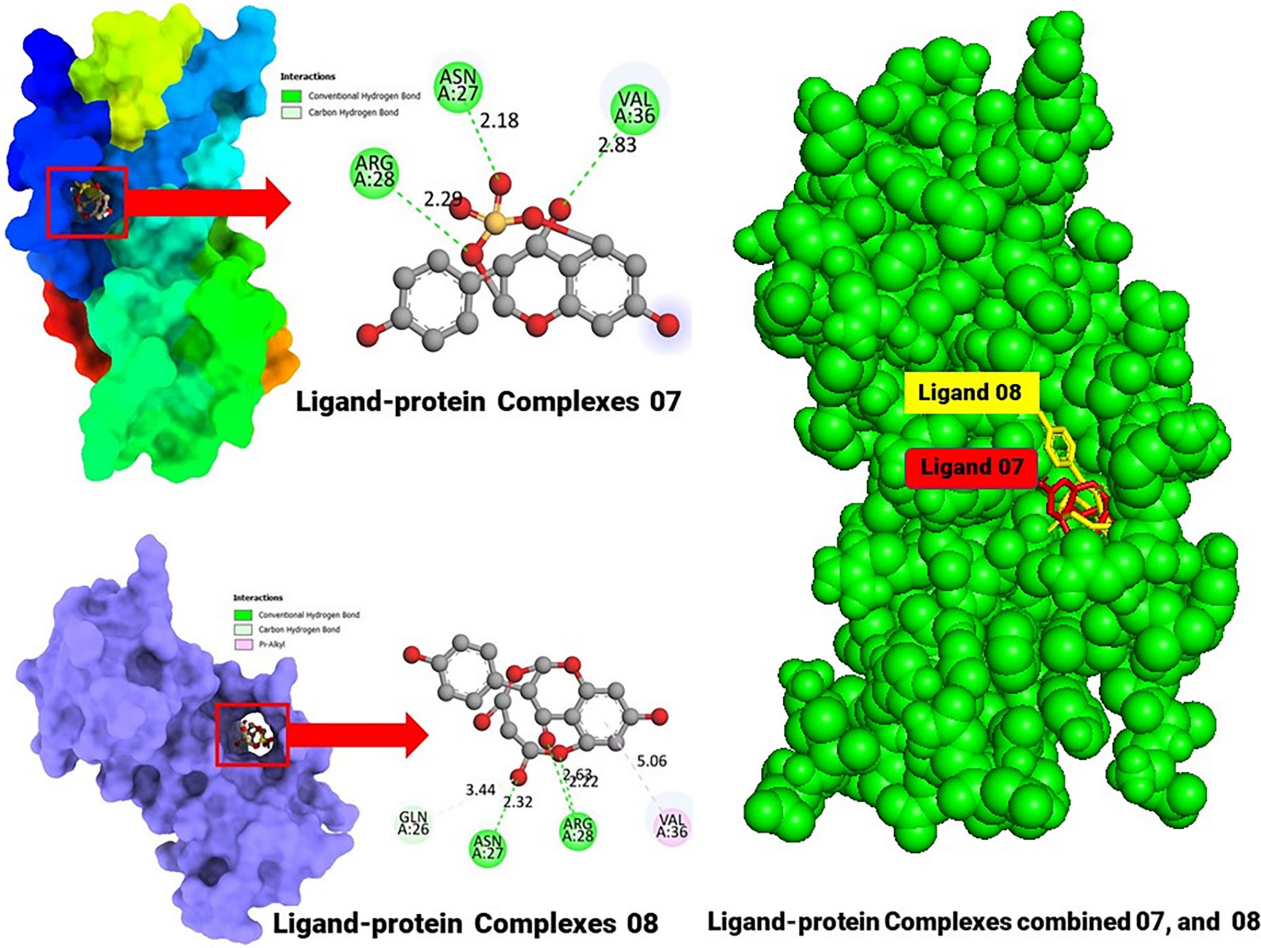
Three docking interactions between the proposed compound.

We then extensively screened the ligand–protein complexes for these two drugs to identify their active amino acid residues and binding sites. In ligand‐protein complex 07, the active amino acids included ASN A: 27, ARG A: 28 and VAL A: 36, each forming different binding angles. In ligand‐protein complex 08, the active amino acids were identified as ASN A: 27, ARG A: 28, GLN A: 26 and VAL A: 36. Finally, the position of the two docking complexes was examined, and it was found that both are formed bonds near a similar active site (Figure 3).

### 3.6 HOMO‐LUMO calculations and chemical descriptors analysis

Based on the maximum binding energy score, top nine ligand investigated the chemical descriptor analysis and shows the calculated values of HOMO and LUMO values, energy gap, hardness and softness, for all compounds (Table 5). These values were calculated using the DFT/B3LYP method with a 6‐31G basis set. The HOMO‐LUMO energies are essential factors in determining the chemical reactivity of molecules, as explained by the principles of frontier molecular orbital theory. The HOMO‐LUMO gap correlates with the chemical hardness, softness, chemical potential and electrophilic index of each molecule. A greater energy difference between the HOMO and the LUMO contributes to increased kinetic stability and reduced chemical reactivity in a compound. Conversely, a narrow HOMO‐LUMO gap signifies decreased chemical stability, as it allows for the easy transfer of electrons between an elevated LUMO and a lowered HOMO in chemical reactions.^58^

**TABLE 5:** Chemical properties of reported compounds.

PubChem CID	I ≈ −ε_HOMO_	A ≈ −ε_LUMO_	Energy GAP = ε_HOMO_ − ε_LUMO_	Hardness $\left(\eta \right)=\frac{1-A}{2}$	Softness $\left(\sigma \right)=\frac{1}{\eta }$
23955874	−5.702	−1.569	4.133	2.066	0.483
146682925	−5.736	−1.600	4.136	2.068	0.483
139586195	−5.674	−1.565	4.109	2.054	0.486
139588458	−5.679	−1.526	4.153	2.076	0.481
146682926	−5.693	−1.553	4.140	2.070	0.483
14440223	−6.119	−4.882	1.237	0.618	1.616
129661094	−5.006	−6.167	1.161	0.580	1.722
129664277	−6.176	−1.189	4.276	2.138	0.467
46780552	−5.714	−1.655	4.058	2.029	0.492

Upon analysis, it was found that compound CID: 14440223 and 129661094 exhibited the smallest energy gap (ΔE) compared to the other compounds. This observation indicates a higher degree of chemical reactivity and substantial intramolecular charge transfer between an electron‐donating group and an electron‐accepting group. While it is reported that the HOMO‐LUMO gaps for the examined compounds fall within the range of 4.27 eV–1.16 eV (Table 5).

Additionally, Table 5 provides numerical values for the softness parameter. The elements with higher softness values described to degrade more rapidly, as they are less time required to changes in their electron configuration. Conversely, hardness is another essential parameters of a molecule. Generally, compounds with higher hardness values exhibit greater resistance to changes in their electron configuration. The values for hardness and softness are inversely related, with the softness values being significantly higher than the hardness values for all compounds except the CID: 14440223 and 129661094 compounds. Compounds CID: 14440223 and 129661094 demonstrate relatively high softness values, suggesting that their degradation may occur rapidly compared to other compounds. The HOMO‐LUMO diagram is displayed in Figure S1.

### 3.7 Molecular dynamic simulation

In addition to the computational methodologies mentioned earlier, we conducted molecular dynamics (MD) simulation studies to investigate the optimal interactions between ligand–protein complex CID: 129661094 (07), 129664277 (08) and Standard Amoxicillin when bound to the protein. These simulations aimed to assess the stability of these interactions over a specified time period.^59^ The investigation primarily focused on evaluating the stability of the protein–ligand complexes throughout the simulation period by analysing the RMSD of the protein backbone, complex structure and ligand structure. Furthermore, we examined MD simulation trajectories to assess various parameters, including RMSF, Rg, H‐bond analysis, PCA and DCCM. Here, we provide a comprehensive description of the findings for each parameter.

#### 3.7.1 Root mean square deviation (RMSD)

**FIGURE 4:**
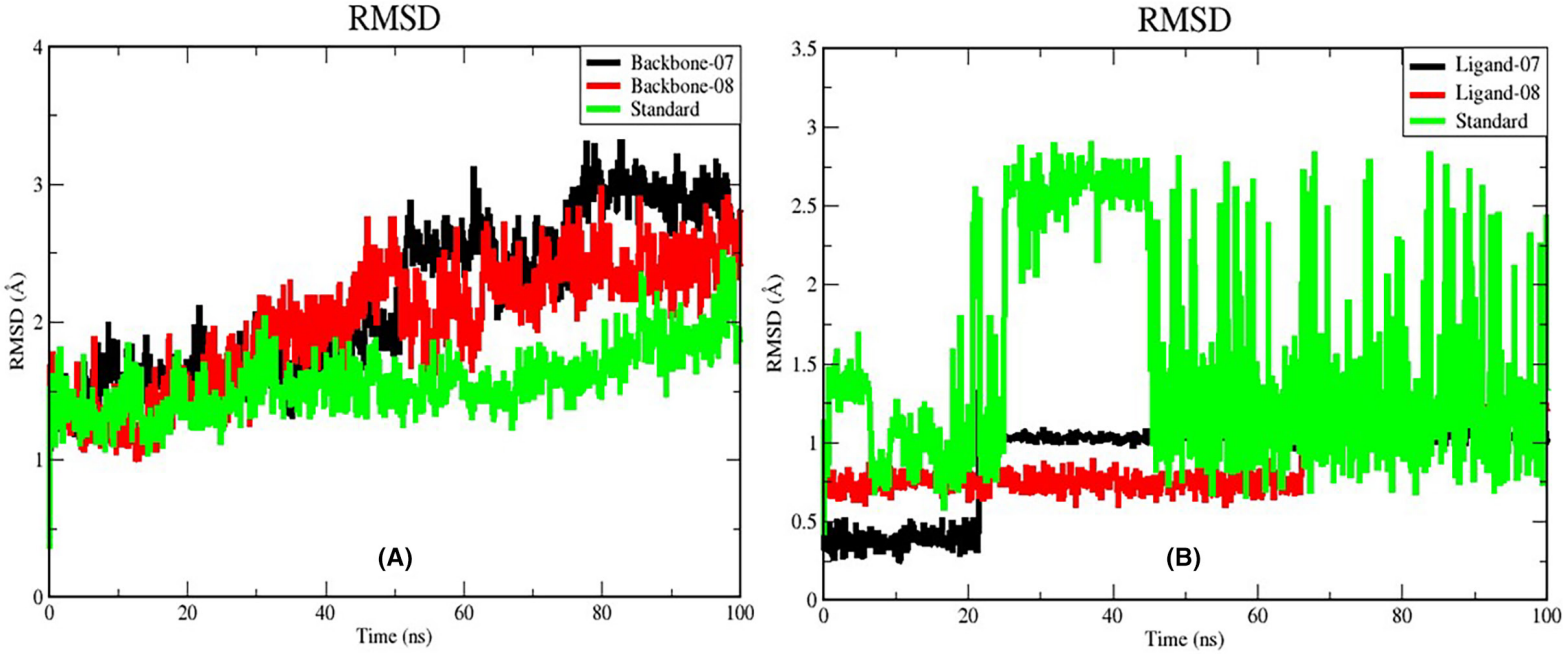
RMSD of (A) backbone and (B) ligands of selected complexes.

To evaluate the stability, we plotted the RMSD values of the backbone atoms from the trajectories. It illustrated the consistent dynamics of all complexes during a simulation period of 100 nanoseconds. Figure 4A shows the RMSD values of the backbone of ligand–protein complex 07 (black), the backbone of ligand–protein complex 08 (red) and the standard backbone (green). In all three complexes, the backbone atoms have moved further away from their starting positions at the beginning of the simulation. This increase was more pronounced in complexes 07 and 08. Between 25 ns and 60 ns, the RMSD values of Backbone 07 and 08 gradually increased, while the RMSD value of the standard system remained stable. In particular, at 50 ns, the RMSD value of system 08 shows an increase of 2.5 Å, while compound 07 shows an increase of more than 2.5 Å after 50 ns. After 60 ns of the simulation, the Backbone complex 08 stabilized with minimal fluctuations until the end of the simulation. The RMSD value of the standard system, on the other hand, continued to rise after 80 ns until the end of the simulation. The average RMSD values for all three compounds are 2.240 Å, 2.058 Å and 1.607 Å respectively.

Figure 4B illustrates the calculation of RMSD for the ligand–protein complex 07, 08 and the standard ligand when they form complexes with (PDB ID:3GUQ). The average RMSD value for ligand‐07 was determined to be 1.415 Å. In contrast, the average RMSD values for ligand‐08 and the standard were determined to be 1.0873 Å and 1.928 Å respectively. In the case of ligand‐07, its stability is high for the first 20 ns of the simulation. Despite significant increase in the RMSD value at 20 ns, it subsequently reached a state of stability and maintained a consistent pattern until the end of the simulation. This observation indicates that the intermolecular interactions of compound ligand‐07 remained intact throughout the simulation. ligand‐08 exhibited analogous characteristics to ligand‐07 during the first 20 ns, and subsequently maintained a considerable degree of stability until around 65 ns. Despite a 1.5 Å rise in the RMSD value at 65 ns, it remained constant throughout the simulation and did not deviate from the binding site.

For the standard ligand, there was an initial rise in the RMSD value during the first 10 ns of the simulation, followed by a subsequent decline in the RMSD value after 10 ns. However, after 20 ns of the simulation, the RMSD value reached 2.5 Å and was observed to leave the binding site. In the last 60 ns of the simulation, the conventional ligand exhibited significant instability and showed the least strong interaction with the target protein.

Based on the results of the RMSD analysis performed for the ligands, it appears that ligand‐protein complex 08 remains constant in its conformational orientation without any significant deviation, while the standard ligand in particular undergoes very significant changes in its binding orientation during the simulation.

#### 3.7.2 Root mean square fluctuation (RMSF)

The RMSF measures how far the atomic sites have deviated from the starting point. The RMSF values of the residues in the C‐ and N‐terminal regions show a considerable degree of fluctuation in all complexes. Ligand‐Protein complex 07 and ligand‐protein complex 08 in particular show greater fluctuations compared to other regions within the protein structures. The residues located in these specific regions of the protein structure are commonly referred to as termini or tails. Consequently, they have a higher degree of mobility and show higher reactivity.

**FIGURE 5:**
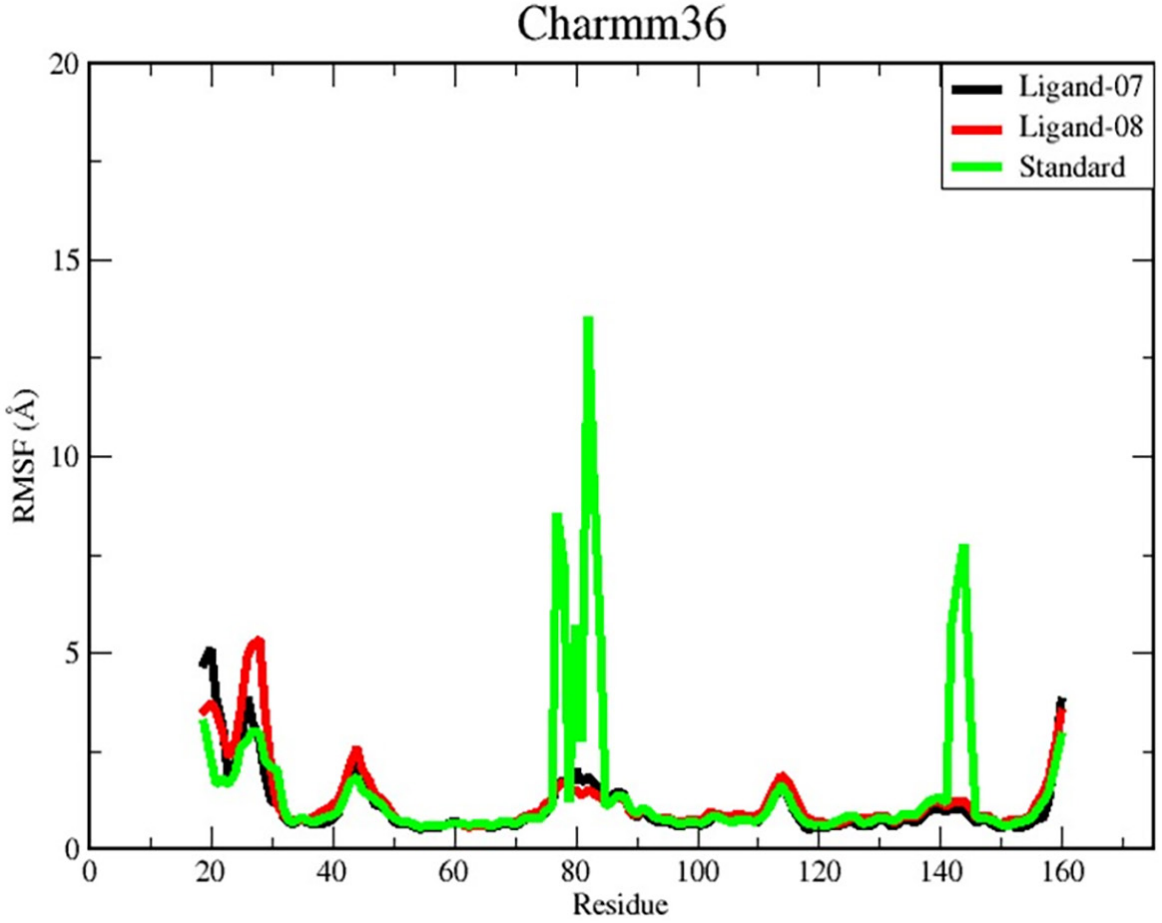
The graphs display RMSF values for three compounds complexed with the target receptor *Helicobacter pylori*‐carcinogenic TNF‐alpha‐inducing (PDB ID: 3GUQ) during 100 ns MDS assessments. The selected drug candidate compounds, Ligand 07 and Ligand 08, along with the standard drug associated with the protein, are represented by black, red, and green colours respectively.

The RMSF chart produces a graphical representation characterized by distinct peaks, with each peak corresponding to a relatively high RMSF value (Figure 5). A total of five peaks were identified in the ligand‐protein complex 07. These peaks were located between the 26th and 27th amino acid residues, between the 43rd and 46th amino acid residues, and between the 77th and 81st, 112th and 115th, and 141st and 144th amino acid residues. Similarly, five prominent peaks were observed in the ligand‐protein complex 08 between the 27th and 29th, 43rd and 45th, and between the 77th and 83rd, 112th and 116th, and 140th and 144th amino acid residues.

However, the standard complex, like the other two complexes, has five peaks, although the peaks detected between residues 77–83 and 142–145 are much more pronounced compared to the other two complexes. In this analysis, we assume that the ligand–protein interactions of the standard complex have less stable binding, which is reflected in the higher RMSF values.

#### 3.7.3 Radius of gyration (Rg) analysis

To evaluate the changes in structural compression, the diagram of the radius of gyration of each structure was monitored throughout the simulation. The compactness of the system was assessed by calculating the Rg, with higher Rg values indicating lower compactness (more unfolded) with increased conformational entropy and lower Rg values indicating higher compactness with greater structural stability (more folded).

**FIGURE 6:**
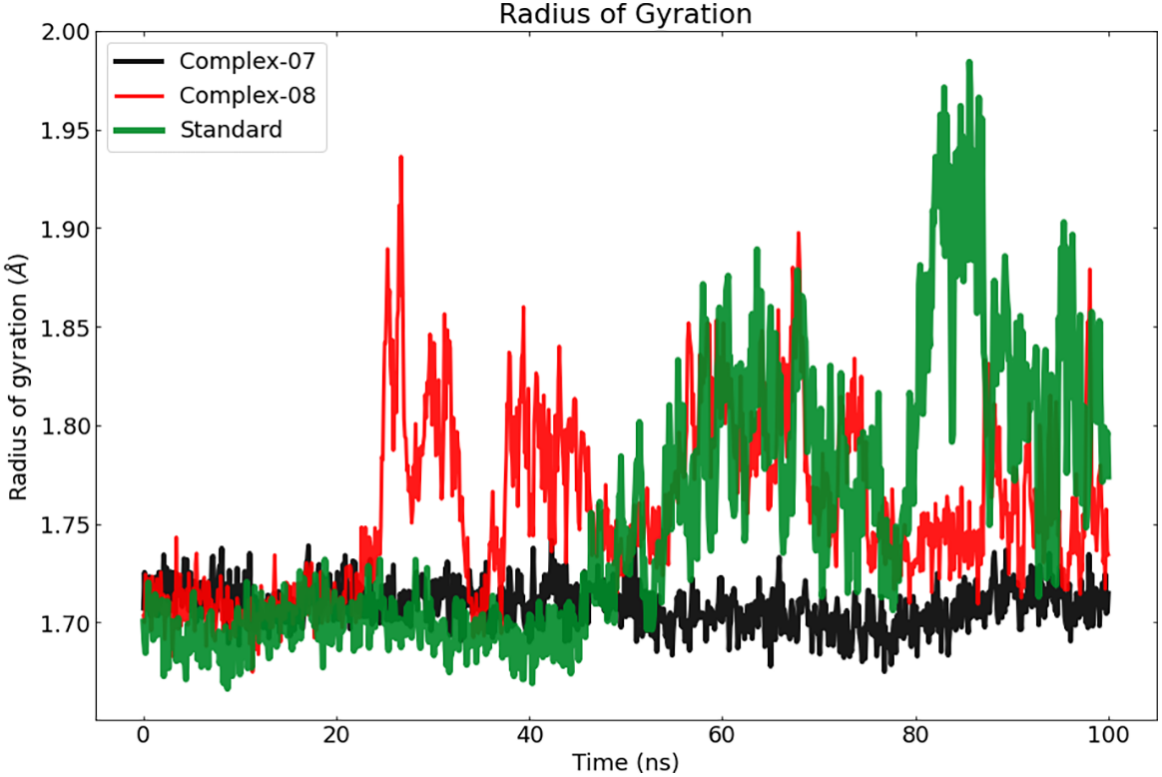
Radius of gyration (Rg) graph for the 100 ns MDS of complex‐07, complex‐08 and standard.

Figure 6 shows that ligand‐protein complex‐07 exhibits minimal changes and maintains stability throughout the simulation period. In contrast, complex‐08 maintains its initial compactness until about 23 ns. Thereafter, the compactness increases significantly between 23 and 37 ns, leading to an Rg value of 1.95 Å. Subsequently, the Rg value increases significantly at the 40 ns mark, in the interval from 50 to 70 ns and during the last 10 ns, although it drops sharply to about 1.70 Å throughout the simulation. In the case of the standard complex, the structure shows only minimal fluctuations up to 45 ns; only an increase in the Rg value can be observed between 45 and 70 ns. At 85 ns, the Rg value reaches its maximum within the simulation. Although the Rg value subsequently decreases, it fluctuates up and down until the end of the simulation and does not reach equilibrium.

Overall, the observed fluctuations in Rg values show different patterns for each complex. Based on the results, Complex‐07 exhibits the smallest Rg value, indicating a greater possibility of compactness compared to the other complexes, which in turn implies a higher affinity for binding between the ligand and the active site. The standard complex, on the other hand, has the highest RG value and was reported to undergo changes indicating a less tightly bound structure of the complex.

#### 3.7.4 Hydrogen bond analysis

During the simulation, in addition to analysing RMSD, RMSF and Rg, we performed an assessment of the stability of the hydrogen bonds (H‐bonds) within the protein–ligand complexes. To gain a comprehensive understanding of the intermolecular relationships between biomolecules, it is essential to perform a detailed geometric analysis of the hydrogen bonds. Hydrogen bonds play a central role in maintaining the stability of complexes in MD modelling.^60^, ^61^, ^62^

Analysis of hydrogen bond occupancy using the Visual Molecular Dynamics (VMD) tool identified all possible hydrogen bonding interactions between the protein and the ligand over time, their stability and relative abundance (Table 6).^63^ The cut‐off values for the distance and angle of the hydrogen bonds (donor H. and acceptor) were set to 3.0 Å and 20° respectively.

**TABLE 6:** Analysis of H‐bond occupancies for each complex during MD simulation.

	Donor acceptor	Occupancy
Ligand‐07	TRP‐23	29.74%
	GLN‐34 UN	10.18%
	GLN‐34	11.88%
Ligand‐08	ASN‐65	5.39%
	TYR‐31	4.39%
	GLU‐117	5.49%
Standard	GLU72	6.59%
	GLU‐72	4.89%
	SER‐29	9.78%

Docked complexes exhibit a higher degree of stability in hydrogen bond formation. During the MD simulations, the hydrogen bonds present in the docking structures were not only retained, but additional hydrogen bonds were also discovered. The ligands ligand‐07 and ligand‐protein complex 08, as well as the standard molecules each show different occupancies of the recognized hydrogen bonds, which are documented in Table 6.

The stability of the ligand‐protein complex‐07 was maintained by interactions with residues TRP 23 and GLN34 residues. The hydrogen bonding between ligand‐07 and TRP 23 has a maximum occupancy of 29.74%. The occupancy is 10.18%, with ligand‐07 acting as a donor for GLN 34 and as an acceptor with GLN 34 at 11.88%. In addition, the ligand‐protein complex 07 formed only three hydrogen bonds as observed in the docked simulations. The complex‐08 has a total of 42 hydrogen bonds, with a maximum occupancy of 5.49%. It is noteworthy that the hydrogen bonds are formed between ligand‐08 and GLU 117. Of the total 42 hydrogen bonds, only four were successfully formed during the docking simulation. From our analysis, it can be deduced that complex 08 exhibits greater stability during the MD process than complex 07. The standard complex has a total of 53 hydrogen bonds, with the highest occupancy being 9.78%. In this complex, the standard molecule acts as a hydrogen bond donor, while SER 29 acts as a hydrogen bond acceptor. The occupancy is 6.59% when the standard molecule acts as an acceptor for GLU 72 and 4.89% when GLU 72 is present. Of the total 53 hydrogen bonds, only three were successfully generated during the docking simulation.

During the first 40 ns of the simulation, there was a considerable formation of hydrogen bonds in both the complex‐08 and the standard system. The complex‐08 system in particular showed the highest level of hydrogen bond formation. After 40 ns, the formation of hydrogen bonds decreased in both the complex‐08 and standard systems. However, in the complex‐07 system, the formation of hydrogen bonds increased throughout the simulation. Conversely, ligand‐07 has a lower total number of hydrogen bonds compared to ligand‐08, but has a higher hydrogen bond occupancy. The stability of each system is determined by the total number of hydrogen bonds and the utilization of these hydrogen bonds. The number and presence of hydrogen bonds play a crucial role in stabilizing the bond between the protein and the ligand complex (Table 6).

#### 3.7.5 Principal component analysis (PCA)

Principal component analysis (PCA) was employed to analyse the dynamics movement within the receptor–ligand complex during a 100‐ns simulation.^64^, ^65^, ^66^ The results were presented as eigen fractions, representing the proportion of variance, and were obtained from a covariance matrix comprising 20 eigen models. Each complex system underwent PCA computations using three conformations, specifically PC1, PC2 and PC3, via standard molecular dynamics (Figure 8A). The findings from the principal component analysis indicated conformational changes within all clusters. Notably, the blue region exhibited the most pronounced movements, the white region displayed intermediate motions and the red region presented the least flexible movements.

Comparing the PCA plot of ligand‐07 (Figure 8A), ligand‐08 (Figure 8B) and standard (Figure 8C), the PC1 cluster has 39.19%, 36.47% and 21.33% of the variance, correspondingly, the PC2 cluster has 11.52%, 20.92% and 10.03% of the variance, while the PC3 cluster has 7.72%, 6.82% and 8.37% of the variance for each system accordingly. Upon examining the variations of PC1, it is evident that the ligand‐07 complex displays the biggest variability, as it possesses the highest value. Conversely, the ligand‐protein complex 08 system exhibits the lowest variance (6.82%) in comparison to the other complexes, suggesting minimal conformational alterations.

**FIGURE 7:**
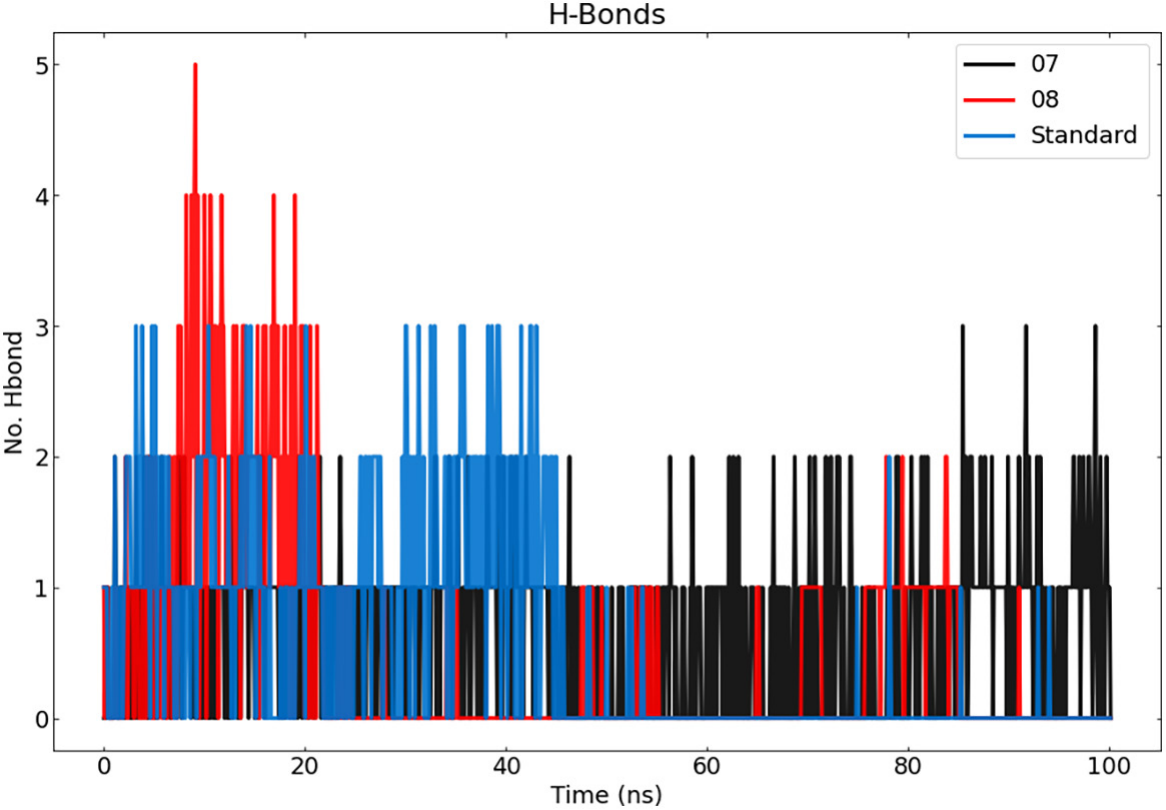
Represents the number of hydrogen bonds responsible for the stability of the complexes (ligand‐07, ligand‐08 and standard) throughout the 100 ns.

**FIGURE 8:**
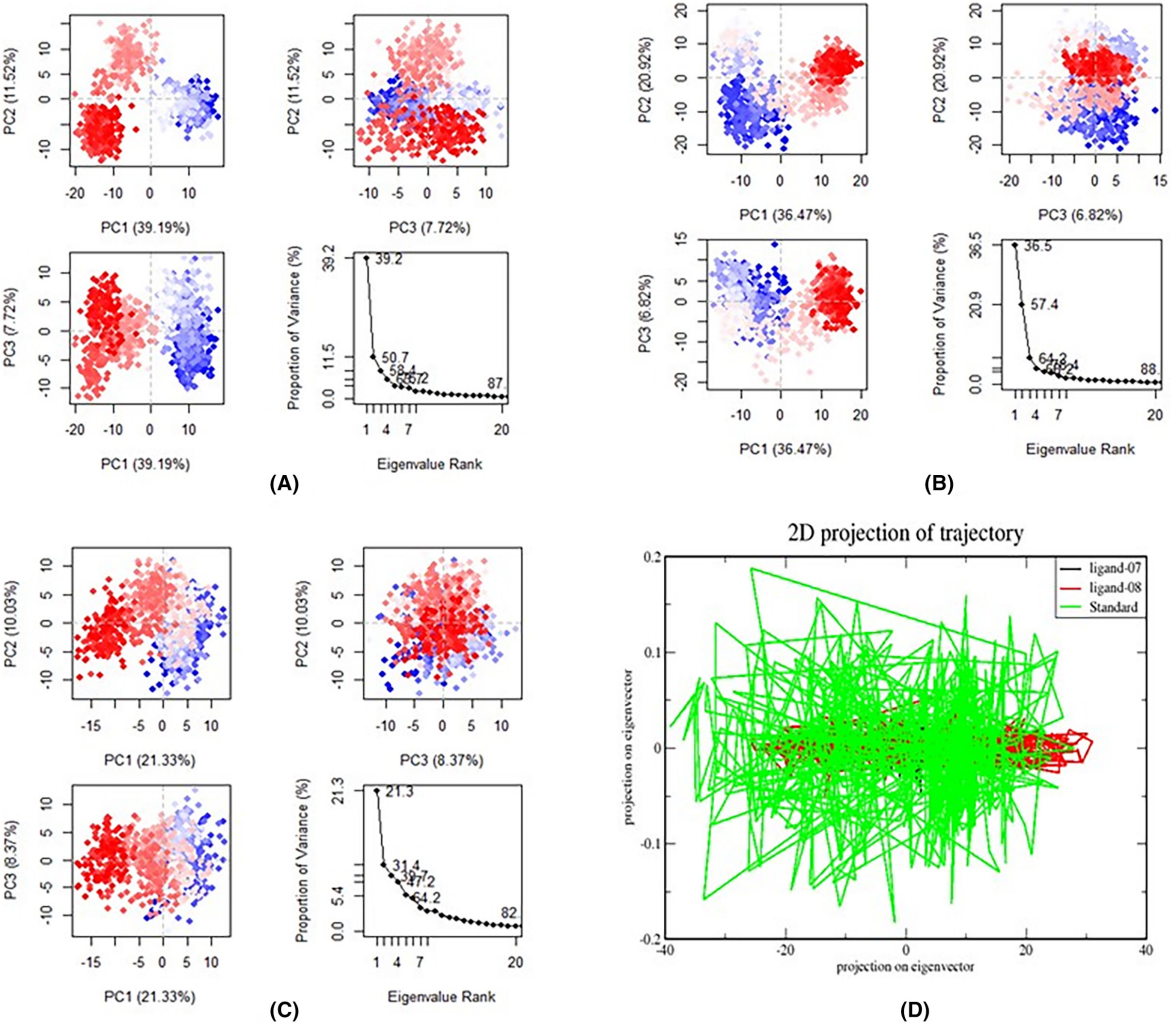
Principal component analysis of (A) protein backbone for ligand‐07, (B) protein backbone for ligand‐08, (C) protein backbone for standard and (D) Dynamics of protein–ligand interactions 2D projections of trajectories on eigenvectors of ligands‐07, ligand‐08 and standard bound proteins.

In addition, we explored the kinetics of protein‐ligand interactions by creating a two‐dimensional projection graph using PCA. We analysed the movements by utilizing the initial two principle components, PC1 and PC2. Figure 7A depicts the exploration of different conformations of protein–ligand complexes in a crucial subspace, using ligands 7, ligand‐08 and a standard ligand. In the 2D projection plot, the stable cluster is represented by the complex that occupies a smaller phase space, while the non‐stable cluster is depicted by the complex that occupies a larger space (Figure 8D).^20^

#### 3.7.6 Dynamic cross‐correlation matrix (DCCM) analysis

In addition, the dynamic cross‐correlation plots obtained showed both positive and negative effects of amino acid correlation. The DCCM values were used to represent the overall correlation, with a range from −1.0 to 1.0, corresponding to colours from dark purple to dark blue. Different colours were used to represent different levels of linkage between the residues, with darker colours indicating a stronger association. Correlations approaching a value of 1 indicate that the residues are moving in the same direction, while correlations approaching a value of −1 indicate movement in opposite directions.^67^

To visualize the association between the indices of the I and J residues, a series of pairwise correlation charts were generated. The expected map results were interpreted using colours such as dark cyan, white and pink. Pairs that showed complete correlation were visually represented with the colour cyan, and pairs that showed anti‐correlation were marked with by pink.

**FIGURE 9:**
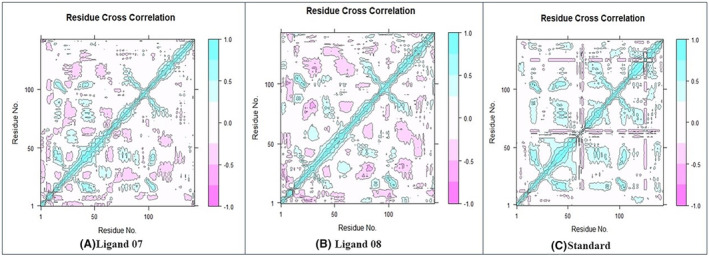
(A) Dynamic cross‐correlation matrix (DCCM) analysis of Cα atoms for the protein–ligand‐07 system. (B) DCCM analysis of Cα atoms for the protein–ligand‐08 system and (C) DCCM analysis of Cα atoms for the protein‐ligand standard system.

Compared to the ligand‐08 system, the full ligand‐protein complex 07 exhibited a significant increase in collective movements with positive correlations, accompanied by a notable increase in movements with negative correlations. These significant changes were observed in the movements with both positive and negative correlations of the full ligand‐07 and ligand‐08 systems compared to the standard system (Figure 9A–C).

#### 3.7.7 Binding free energy and ligand‐residue interaction decomposition

A comprehensive investigation using the molecular mechanics Poisson‐Boltzmann surface area (MM‐PBSA) method was performed to determine the binding free energy and analyse the molecular interactions and stability between ligand‐protein complex 07, ligand‐protein compex 08 and standard with the carcinogenic TNF‐alpha‐inducing target protein of *Helicobacter pylori* (PDB ID: 3GUQ). This analysis provides valuable insights into the intricate mechanisms underlying its binding and stability.

Additionally, it provides a representation of the residue decomposition that helps to investigate the contribution of the amino acid residues involved in the spatial interactions that stabilize the ligands in the binding pocket of the protein.^68^

**FIGURE 10:**
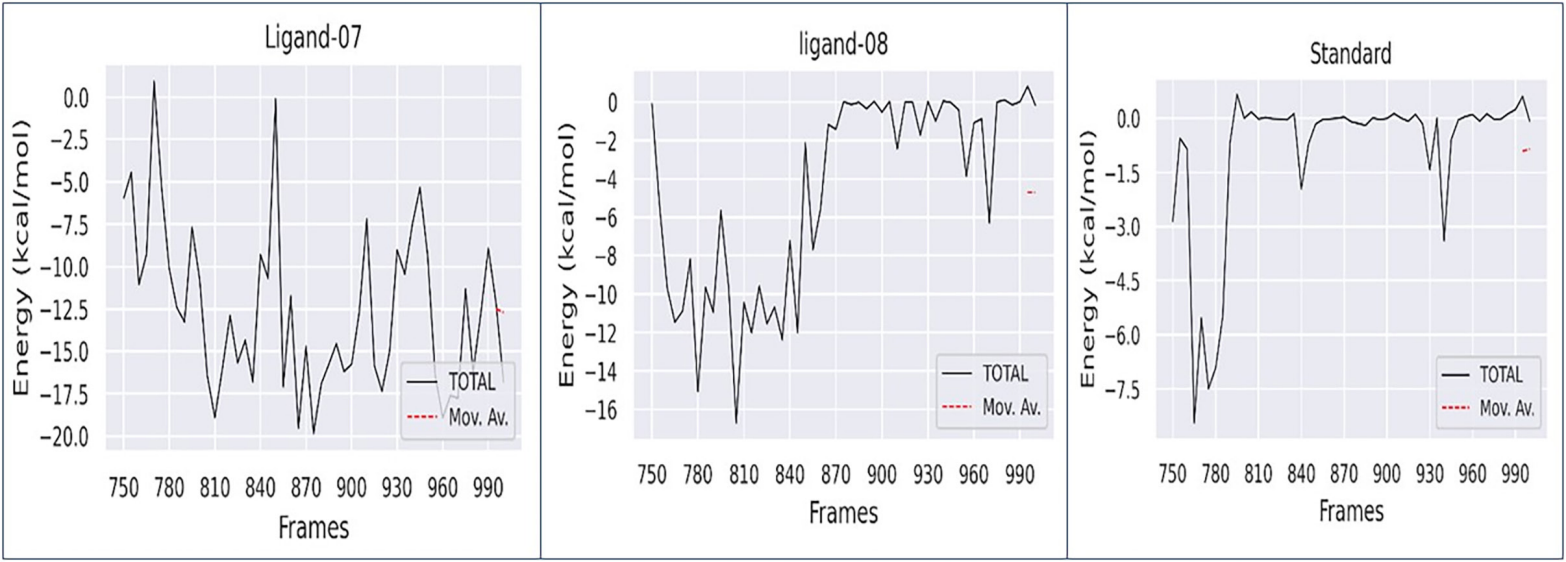
Binding free energy plot graphically displayed. First one ligand–protein complex 07, middle one ligand protein complex 08 and the last one is ligand–protein complex standard.

The more negative the values, the more favourable the binding free energy between proteins and ligands. Table 7 and Figure 10 display the free binding energies of the ligands and correspond to the outcomes are −12.58 ± 1.18 kJ/mol for complex 07, −4.63 ± 3.78 kJ/mol for complex 08 and − 0.91 ± 2.75 kJ/mol for complex standard. These means that complex 07 has the strongest binding free energy compare to the complex 08, and standard.

**TABLE 7:** Binding free energy results.

Compounds	ΔEVDW (kJ/mol)	ΔEEEL (kJ/mol)	ΔGPB (kJ/mol)	ΔGNP (kJ/mol)	ΔGDISP (kJ/mol)	ΔG Binding (kJ/mol)
Ligand‐07	−20.81	−12.81	23.59	−2.54	0.0	−12.58 ± 1.18
Ligand‐08	−4.84	−9.79	10.77	−0.77	0.0	−4.63 ± 3.78
Standard	−1.61	−1.46	2.41	−0.25	0.0	−0.91 ± 2.75

**FIGURE 11:**
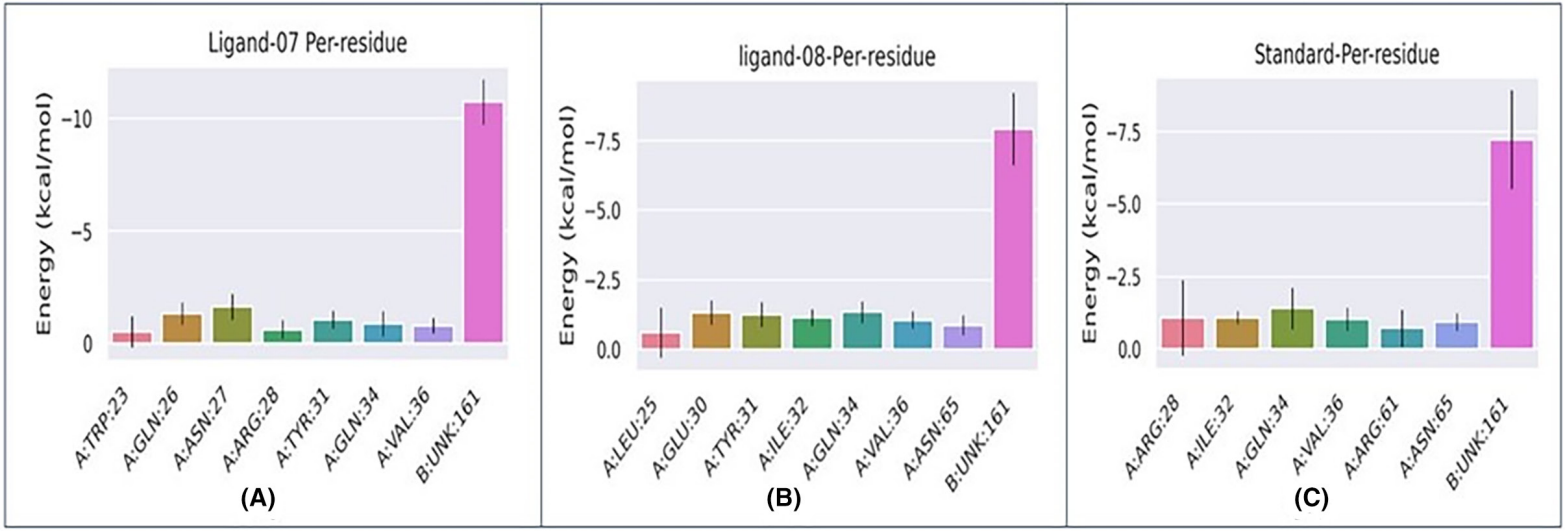
Plot of MM/PBSA binding free energy contribution per residue of each complex.

To better understand the binding process, the MM/PBSA technique was used to decompose the binding free energy into the individual contributions of each residue. The free energy decomposition study shows that the active site residues, including ARG28, ILE32, GLN34, VAL36, ARG61 and ASN65, exhibit a favourable energetic preference for improving the binding stability of the ligand–protein complex‐07 to the protein (Figure 11A). Significantly, ASN27 was found to make the largest contribution to the free energy contribution. Analysis of the binding interaction between ligand–protein complex‐08 revealed that many amino acid residues (Figure 11B), namely LEU25, GLU30, TYR31, ILE32, GLN34, VAL36 and ASN65, made the largest contribution to the overall ΔGbind value, which was measured to be −4.63 ± 3.78.

Figure 11C illustrates the free energy decomposition plot of the standard protein system, highlighting the significant energetic contributions from specific amino acids, namely ARG28, ILE32, GLN34, VAL36, ARG61 and ASN65, in stabilizing the standard molecule within the binding pocket.

## 4 CONCLUSION

The aim of this study was to identify potentially bioactive compounds from the daidzein derivatives as inhibitors of *H. pylori‐associated* gastric cancer. We applied a wide range of computational techniques to evaluate the pharmacological efficacy, and activities of these derivatives. The ligand–protein complexes CID: 129661094 (07) and 129664277 (08) were identified as potential candidates and showed strong binding interactions (−8.5 kcal/mol and −8.9 kcal/mol) in the compound with the target protein, *Helicobacter pylori*‐carcinogenic TNF‐alpha‐inducing protein. In addition, PCA and DCCM analysed the dynamics movement within the receptor–ligand complex. The PCA results showed that ligand–protein complex CID: 129661094 (07) and 129664277 (08) has a structurally compact and stable conformation. The hydrogen bonds showed the presence of positive interactions between the ligands and certain amino acid residues, which increased the overall stability of the complexes formed between these ligands and proteins. Subsequently, the binding free energy analysis was calculated and confirmed that the ligand‐protein complex CID: 129661094 (07) and 129664277 (08) showed a better outcome compared to the standard. Finally, the result of this study shows that the daidzein derivatives have promising efficacy as inhibitors of *H. pylori*, which is closely associated with the development of gastric cancer. Although this computational research provides useful outcomes against *H. pylori* infections. However, it is suggested that further experimental studies be conducted to evaluate the actual efficacy on a large scale.

## AUTHOR CONTRIBUTIONS

**Jehad Zuhair Tayyeb:** Conceptualization (equal); data curation (equal); investigation (equal); methodology (equal); visualization (equal); writing – original draft (lead). **Shibam Mondal:** Conceptualization (equal); data curation (equal); formal analysis (equal); validation (equal); visualization (equal); writing – original draft (equal). **Md Anisur Rahman:** Investigation (equal); resources (equal); software (equal); validation (equal); writing – original draft (equal). **Swapon Kumar:** Conceptualization (equal); formal analysis (equal); methodology (equal); project administration (equal); validation (equal); writing – original draft (supporting). **Imren Bayıl:** Formal analysis (equal); investigation (equal); methodology (equal); project administration (equal); resources (equal); software (equal); validation (equal); visualization (equal); writing – review and editing (equal). **Shopnil Akash:** Conceptualization (equal); investigation (equal); software (equal); validation (equal); writing – original draft (equal). **Md. Sarowar Hossain:** Data curation (equal); formal analysis (equal); investigation (equal); methodology (lead); software (equal); supervision (equal); writing – review and editing (equal). **Taha Alqahtani:** Data curation (equal); supervision (equal); validation (equal); writing – review and editing (lead). **Magdi E. A. Zaki:** supervision (equal): Writing – review and editing (equal). **Jonas Ivan Nobre Oliveira:** Investigation (equal); project administration (equal); resources (equal); software (equal); writing – review and editing (equal).

## FUNDING INFORMATION

No funding.

## CONFLICT OF INTEREST STATEMENT

The authors declare no conflicts of interest.

## Supporting information


Appendix S1.

## Data Availability

The data sets used and/or analysed during this study are available from the corresponding author upon reasonable request.
