# Supplementary material for: Identification of Helicobacter pylori‐carcinogenic TNF‐alpha‐inducing protein inhibitors via daidzein derivatives through computational approaches
Source: J Cell Mol Med. 2024 May 2;28(9):e18358. doi: 10.1111/jcmm.18358 (PMC11063725; doi:10.1111/jcmm.18358)
Supplement: Supplementary file 1 — Appendix S1. [file JCMM-28-e18358-s001.docx]

| **Supplementary Table S1:** Pass prediction spectrum | | | | | | | | | | |
| --- | --- | --- | --- | --- | --- | --- | --- | --- | --- | --- |
| **No** | **PubChem ID** | **Antibacterial** | | **Antiviral (Influenza)** | | | **Antineoplastic** | | **Antiparasitic** | |
|  |  | **Pa** | **Pi** | **Pa** | **Pi** | | **Pa** | **Pi** | **Pa** | **Pa** |
|  | 5281708 | 0.355 | 0.042 | 0.296 | 0.092 | | 0.646 | 0.036 | 0.327 | 0.048 |
|  | 131751509 | 0.569 | 0.011 | 0.646 | 0.009 | | 0.784 | 0.014 | 0.379 | 0.036 |
|  | 11316354 | 0.604 | 0.009 | 0.676 | 0.007 | | 0.713 | 0.024 | 0.396 | 0.032 |
|  | 107971 | 0.570 | 0.011 | 0.638 | 0.010 | | 0.783 | 0.014 | 0.387 | 0.034 |
|  | 136419 | 0.280 | 0.068 | 0.251 | 0.131 | | 0.653 | 0.034 | 0.251 | 0.074 |
|  | 5281807 | 0.522 | 0.014 | 0.664 | 0.008 | | 0.807 | 0.011 | 0.316 | 0.051 |
|  | 156155 | 0.586 | 0.010 | 0.435 | 0.036 | | 0.782 | 0.014 | 0.368 | 0.038 |
|  | 176907 | 0.228 | 0.096 | 0.345 | 0.066 | | 0.657 | 0.034 | 0.237 | 0.080 |
|  | 4183640 | 0.570 | 0.011 | 0.638 | 0.010 | | 0.783 | 0.014 | 0.387 | 0.034 |
|  | 23930394 | 0.604 | 0.009 | 0.676 | 0.007 | | 0.713 | 0.024 | 0.396 | 0.032 |
|  | 12114463 | 0.457 | 0.021 | 0.214 | 0.182 | | 0.258 | 0.180 | - | - |
|  | 10359753 | 0.356 | 0.042 | -- | ---- | | 0.681 | 0.029 | --- | ---- |
|  | 23955874 | 0.606 | 0.008 | 0.551 | 0.017 | | 0.808 | 0.011 | 0.527 | 0.013 |
|  | 171292 | 0.569 | 0.011 | 0.646 | 0.009 | | 0.784 | 0.014 | 0.379 | 0.036 |
|  | 183927 | 0.628 | 0.007 | 0.354 | 0.063 | | 0.855 | 0.006 | 0.209 | 0.093 |
|  | 4118326 | - | - | - | - | | 0.457 | 0.085 | - | - |
|  | 49862229 | 0.570 | 0.011 | 0.638 | 0.010 | | 0.783 | 0.014 | - | - |
|  | 146682925 | 0.622 | 0.008 | 0.359 | 0.061 | | 0.840 | 0.008 | 0.712 | 0.005 |
|  | 10587403 | 0.355 | 0.042 | 0.296 | 0.092 | | 0.646 | 0.036 | 0.327 | 0.048 |
|  | 139586195 | 0.626 | 0.008 | 0.445 | 0.033 | | 0.826 | 0.009 | 0.431 | 0.025 |
|  | 139588458 | 0.633 | 0.007 | 0.507 | 0.022 | | 0.829 | 0.009 | 0.561 | 0.010 |
|  | 146682926 | 0.622 | 0.008 | 0.359 | 0.061 | | 0.840 | 0.008 | 0.712 | 0.005 |
|  | 12241084 | 0.355 | 0.042 | 0.296 | 0.092 | | 0.646 | 0.036 | 0.327 | 0.048 |
|  | 139586577 | 0.325 | 0.051 | 0.215 | 0.175 | | 0.415 | 0.099 | 0.282 | 0.062 |
|  | 146683393 | 0.562 | 0.011 | 0.209 | 0.196 | | 0.781 | 0.014 | 0.423 | 0.026 |
|  | 12114465 | 0.457 | 0.021 | 0.214 | 0.182 | | 0.258 | 0.180 | - | - |
|  | 44257216 | 0.586 | 0.010 | 0.435 | 0.036 | | 0.782 | 0.014 | 0.368 | 0.038 |
|  | 14440223 | 0.217 | 0.103 | - | | - | 0.921 | 0.005 | 0.248 | 0.075 |
|  | 129661094 | - | - | - | | - | 0.917 | 0.005 | - | - |
|  | 129696974 | 0.513 | 0.015 | 0.224 | | 0.154 | 0.672 | 0.031 | 0.232 | 0.082 |
|  | 49862188 | 0.284 | 0.066 | 0.277 | | 0.105 | 0.483 | 0.077 | - | - |
|  | 12241084 | 0.355 | 0.042 | 0.296 | | 0.092 | 0.646 | 0.036 | 0.327 | 0.048 |
|  | 71315932 | 0.207 | 0.111 | 0.244 | | 0.138 | 0.443 | 0.089 | 0.146 | 0.132 |
|  | 129664277 | 0.211 | 0.108 | - | | - | 0.938 | 0.004 | 0.169 | 0.112 |
|  | 139587160 | 0.577 | 0.010 | 0.528 | | 0.019 | 0.445 | 0.089 | 0.239 | 0.079 |
|  | 44257217 | 0.570 | 0.011 | 0.638 | | 0.010 | 0.783 | 0.014 | 0.387 | 0.034 |
|  | 129698225 | 0.350 | 0.043 | 0.299 | | 0.090 | 0.399 | 0.104 | 0.223 | 0.086 |
|  | 167995018 | 0.486 | 0.018 | - | | - | 0.751 | 0.018 | 0.537 | 0.012 |
|  | 46780552 | 0.203 | 0.114 | - | | - | 0.908 | 0.005 | 0.457 | 0.020 |
|  | 73032774 | 0.570 | 0.011 | 0.687 | | 0.006 | 0.788 | 0.013 | 0.325 | 0.049 |
|  | 92024357 | 0.604 | 0.009 | 0.676 | | 0.007 | 0.713 | 0.024 | 0.396 | 0.032 |
|  | 57509377 | 0.570 | 0.011 | 0.687 | | 0.006 | 0.788 | 0.013 | 0.325 | 0.049 |
|  | 131700444 | 0.604 | 0.009 | 0.676 | | 0.007 | 0.713 | 0.024 | 0.396 | 0.032 |
|  | 29976718 | 0.556 | 0.012 | 0.672 | | 0.008 | 0.735 | 0.020 | 0.315 | 0.051 |
|  | 92132082 | 0.466 | 0.020 | 0.296 | | 0.092 | 0.694 | 0.027 | 0.380 | 0.035 |


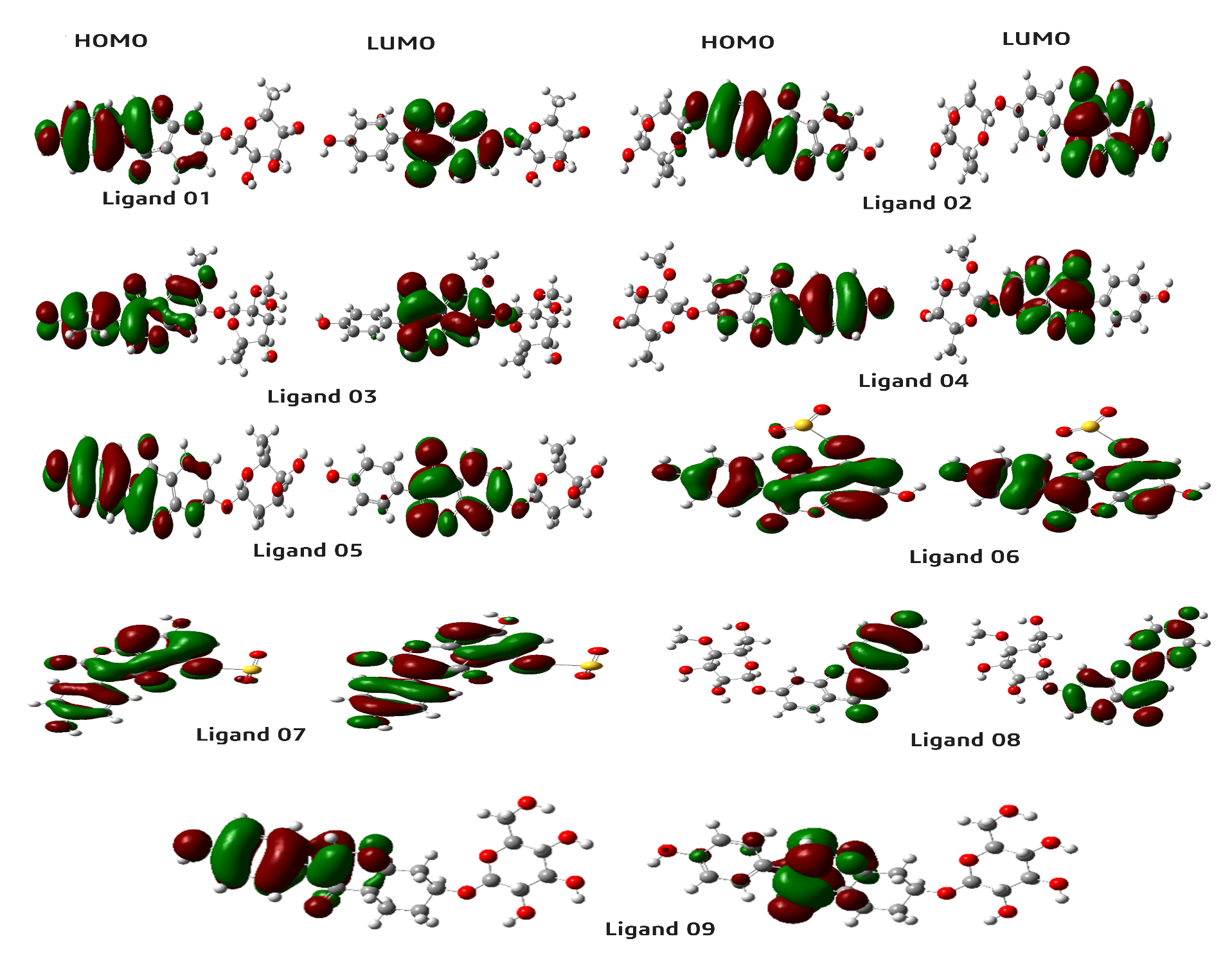
**Supplementary Figure 1.** Diagram of Frontier molecular orbitals
